# [Corrigendum] Identification of Akt1 as a potent therapeutic target for oral squamous cell carcinoma

**DOI:** 10.3892/ijo.2025.5790

**Published:** 2025-08-19

**Authors:** Koh-Ichi Nakashiro, Hiroshi Tanaka, Hiroyuki Goda, Kazuki Iwamoto, Norihiko Tokuzen, Shingo Hara, Jun Onodera, Ichiro Fujimoto, Satoshi Hino, Hiroyuki Hamakawa

Int J Oncol 47: 1273-1281, 2015; DOI: 10.3892/ijo.2015.3134

Following the publication of the above paper, it was drawn to the Editor's attention by a concerned reader that, regarding the western blots shown in Figs. 1C and 3A, the six lanes for the control β-tubulin blots shown in Fig. 1C appeared strikingly similar to the β-tubulin blots in Fig. 3A, albeit the bands in Fig. 3A appeared to have been horizontally stretched.

The authors were able to check their original data, and realized that Fig. 3 had inadvertently been assembled incorrectly. A revised version of Fig. 3, now showing data for both the Akt1 and β-tubulin blots from one of the repeated experiments, is shown opposite. All authors confirm that the errors made in assembling Fig. 3 did not have a major impact on the conclusions reported in the above article, and they thank the Editor of *International Journal of Oncology* for allowing them the opportunity to publish a Corrigendum. Furthermore, all the authors agree to the publication of this Corrigendum, and apologize to the readers for any inconvenience caused.

## Figures and Tables

**Figure 3:**
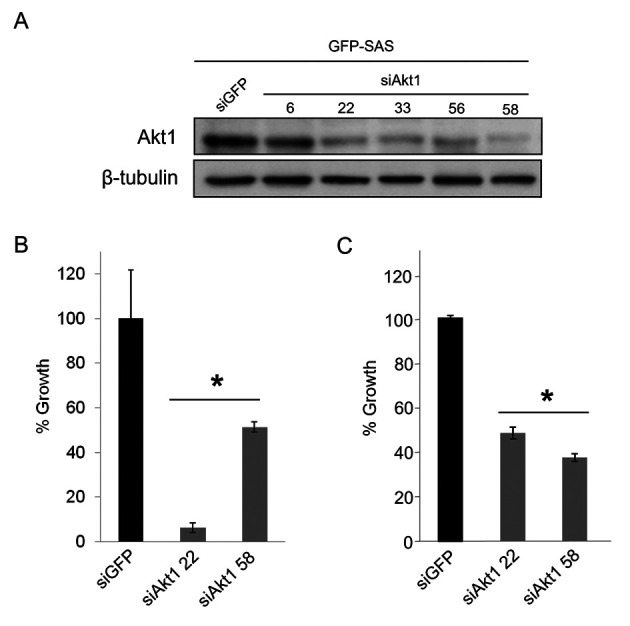
Effects of Akt1 knockdown in the human OSCC cell line, GFP-SAS. (A) Five siAkt1 constructs (siAkt1-6, siAkt1-22, siAkt1-33, siAkt1-56, and siAkt1-58) were transfected into cells at 10 nM with Lipofectamine RNAiMAX. The effects on Akt1 protein expression were evaluated by western blotting and showed that all siAkt1 constructs suppressed expression. (B) The effects of siAkt1-22 and 58 on cell growth were assessed in cells seeded in complete medium and both siRNA constructs significantly inhibited cell growth. (C) The effects of siAkt1-22 and 58 on the invasive growth were examined in the collagen gel culture system and they significantly suppressed the invasive growth. ^*^p<0.01 compared to control cultures transfected with siGFP.

